# Influence of *Lactobacillus paracasei* HII01 Supplementation on Glycemia and Inflammatory Biomarkers in Type 2 Diabetes: A Randomized Clinical Trial

**DOI:** 10.3390/foods10071455

**Published:** 2021-06-23

**Authors:** Parichart Toejing, Nanticha Khampithum, Sasithorn Sirilun, Chaiyavat Chaiyasut, Narissara Lailerd

**Affiliations:** 1 Department of Physiology, Faculty of Medicine, Chiang Mai University, Chiang Mai 50200, Thailand; lookplanoi_@hotmail.com; 2 Innovation Center for Holistic Health, Nutraceuticals and Cosmeceuticals, Department of Pharmaceutical Sciences, Faculty of Pharmacy, Chiang Mai University, Chiang Mai 50200, Thailand; nantichakhampithum@gmail.com (N.K.); sasithorn.s@cmu.ac.th (S.S.)

**Keywords:** *Lactobacillus paracasei*, probiotics, type 2 diabetes mellitus, gut microbiota, glycemia, inflammation

## Abstract

It has been shown that gut dysbiosis can be associated with the development of type 2 diabetes mellitus (T2DM). Consequently, intervention with probiotics may be a useful approach to improve metabolic variables in diabetes. The present study aimed to evaluate the efficacy of *L. paracasei* HII01 on glycemia in T2DM patients. In a randomized, double-blind, placebo-controlled study, 50 participants were allocated to receive *L. paracasei* HII01 (50 × 10^9^ CFU/day) or a placebo (corn starch 10 mg/day). Blood and fecal samples were assessed at baseline and at the end of the trial. After 12 weeks of intervention, fasting blood glucose level had significantly decreased in the probiotic group compared with the placebo group. Importantly, probiotic supplementation significantly decreased the plasma levels of LPS, TNF-α, IL-6 and hsCRP compared the placebo group. Additionally, an increase in beneficial bacteria and a decrease in pathogenic bacteria, which related to the improvement of SCFAs, was found following *L. paracasei* HII01 supplementation. These findings demonstrated that *L. paracasei* HII01 improved hyperglycemia and inflammatory markers by favorably modifying gut microbiota and subsequently ameliorating the leaky gut and endotoxemia, thereby suggesting a potential role as an adjuvant treatment in type 2 diabetes.

## 1. Introduction

Type 2 diabetes mellitus (T2DM) is one of the most common metabolic disorders in the world. In 2019, it was estimated that 463 million people worldwide had diabetes. Moreover, the prediction found that the number of diabetic patients will reach to 578 million in 2030 and 700 million in 2045 [1]. T2DM is characterized by hyperglycemia due to either insulin insufficiency, insulin resistance or both [2]. Diabetes is an established major independent risk factor for several chronic diseases, such as ischemic heart disease, stroke and renal failure, which caused the death of 4.2 million people in 2019 [3]. These diabetic complications can be prevented or reduced by sustained control of blood glucose. Nowadays, drugs in the treatment of diabetes can trigger several serious side effects. Thus, there is still a need for safer and effective hypoglycemic agents.

Gut microbiota have been accepted as a key environmental factor contributing to the development of diabetes [4]. Alteration of gut microbiota (dysbiosis) can disrupt the gut’s tight junctions, leading to increased gut permeability and favoring lipopolysaccharide (LPS) translocation to blood circulation. Increased circulating LPS induces “metabolic endotoxemia,” which triggers inflammatory reactions and insulin resistance [5]. Modulation of gut microbiota via probiotics has been widely used to prevent the development of T2DM and its complications. Probiotics refer to microorganisms that confer health benefits to hosts when administered in adequate amounts [6]. Prebiotics is defined as a selectively fermented ingredient that results in specific changes in the composition and/or activity of the gastrointestinal microbiota, thus conferring benefits upon host health [7]. *Lactobacillus* and *Bifidobacterium* are the strains most commonly used as probiotics in functional foods and dietary supplements [8]. A clinical study demonstrated that treatment with probiotic yogurt (7.23 × 10^6^ CFU of *L. acidophilus* La5 and 6.04 × 10^6^ CFU of *B. lactis* Bb12) for 6 weeks reduced fasting blood glucose and HbA1c levels in T2DM patients [9]. The consumption of *L. plantarum* WCFS1 at a dose of 10^12^ CFU/day increased the tight junction protein, ZO-1, in healthy subjects [10]. Additionally, it has been reported that treatment with *L. acidophilus* La-5 and *B. animalis* subsp *lactis* BB-12 (10^9^ CFU/day, each) decreased the blood level of inflammatory cytokines (TNF-α and resistin) and increased in the SCFAs level of acetic acid in the feces of T2DM patients [11]. Recently, Shaun et al. (2019) found that supplementation with probiotic mixture for 6 months reduced blood LPS level resulting in decreased fasting blood glucose, HOMA-IR, inflammatory markers (TNF-α, IL-6), C-reactive protein (CRP) and resistin in patients with diabetes [12]. However, the actual effects of probiotic supplementation on gut microbiota or glucose metabolism are still under debate in clinical studies.

Recently, a newly identified probiotic *Lactobacillus paracasei* HII01 (*L. paracasei* HII01), from the fermentation of northern Thai pickle, was found to have beneficial effects in an animal model of obesity and diabetes [13,14]. However, the hypoglycemic effect and its potential mechanism of *L. paracasei* HII01 in T2DM patients need to be validated. Therefore, the aim of the present study was to evaluate the efficacy of the probiotic *L. paracasei* HII01 on the treatment of glycemia in T2DM patients. In addition, we focused on the modulation of gut microbiota, gut permeability and its contribution to improving systemic inflammation.

## 2. Materials and Methods

The study protocol was approved by the Ethics Committee of Phrae Provincial Public Health Office (approval number: PPH No.1/2562) and conducted according to the guidelines of the National Policy Guidelines for Human Research 2015, National Research Council of Thailand. The study was performed under the supervision of physicians. Prior to the study, the purpose and methodology of the study were fully explained to the participants by the researchers, and all patients gave written informed consent before any study procedures were initiated.

A randomized, double-blind, placebo-controlled clinical trial was used in this study. in fasting plasma glucose concentrations as the primary outcome and the change of *Bifidobacterium* in feces as the secondary outcome [15]. To detect a 25% decrease in fasting plasma glucose concentrations after the intervention or an abundance of *Bifidobacterium* of the intervention group, which was 7.36 (standard deviation = 0.79) at pretreatment period and 7.94 (standard deviation = 0.87) at post-treatment period, an α value equal to 0.05 and a power of 80% were considered using the STATA program [15]. The maximum number of estimated sample size from each calculation was chosen and used in this study. Considering 20% probable drop in the sample, at least 50 participants were allocated to placebo and intervention groups (25 in each group).

### 2.1. Participants

The eligible participants consisted of individuals (men and women) recruited from health-promoting hospitals in Phrae Province, Thailand. The inclusion criteria were T2DM according to WHO criteria [16], being aged between 20 and 70 years and not having had any antibiotic treatment for 14 days prior to the start of the study to prevent the bactericidal effect. Although antibiotic drugs have a side effect to reduce the diversity of gut microbiome, the recovery of microbiome disturbances after antibiotics depends on many factors such as type of antibiotic drug, dosages, duration of taking or plasma half-life. For example, amoxicillin has been reported to not change total bacterial numbers and microbial diversity significantly [17]. The exclusion criteria were an abnormal liver or renal function test, a history of malignant, micro- and macrovascular complications, chronic alcoholism or heavy alcohol use (defined heavy alcohol use as binge drinking on 5 or more days in the past month) [18], being pregnant or breastfeeding, taking nonsteroidal anti-inflammatory drugs, heavy cigarette smoking or heavy smoker (defined as more than 20 cigarettes daily) [19] and hormone replacement therapy. Additionally, participants were excluded from the study if there was any change in medication or lifestyle, or they started to take antibiotics at any stage of the investigation.

### 2.2. Study Protocol

**Figure 1:**
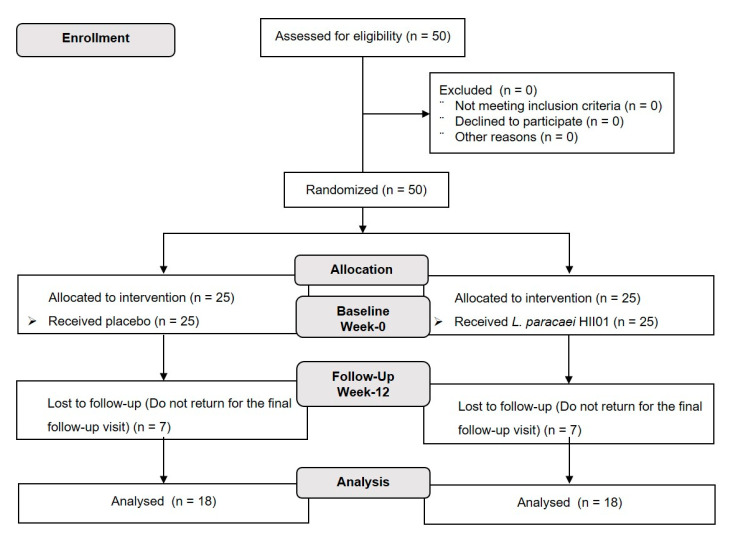
Flowchart of the study design.

The participants (*n* = 50) were randomly assigned by blocked randomization at a ratio of 1:1 with a computer-generated assignment to either probiotic or placebo groups. The investigators, study staff and participants were blinded to the group assignment. Participants in the probiotic group received a probiotic *L. paracasei* HII01 50 × 10^9^ CFU/day and the placebo group received corn starch 10 mg/day throughout the 12 weeks of intervention. The study design is presented in Figure 1. The probiotic *L. paracasei* HII01 was produced by LACTOMASON Company (Gyeongsangnam-do, Korea). Both the probiotic and the corn starch were contained within an aluminum foil envelope. All participants were required to:

- Take one aluminum foil envelope per day (20 min before dinner or sleeping) with clean drinking water.
- Store the probiotic or placebo in the fridge at 4–6 °C.
- Record the number of aluminum foil envelopes taken each day in the study diary.
- Avoid eating or drinking yoghurt, fermented food, dietary supplements (i.e., vitamins, minerals, nutraceuticals, herbal preparations, probiotics, prebiotics or fish oils).

The primary assessment of compliance was evaluated from the count of aluminum foils. In addition, participants’ recording books containing dietary and medication intake, physical activity, defecation and any undesirable side effects were considered.

### 2.3. Outcome Measures

The primary outcome measurements were fasting blood glucose (FBG) and HbA1c concentration at the end of the 12-week study period. The secondary outcomes included bacterial and SCFAs abundance in feces, gut permeability (plasma ZO-1), plasma LPS, plasma IgA, plasma inflammatory biomarkers (IL-1β, IL-6, IL-10, TNF-α and hsCRP), plasma lipid (TG, cholesterol, HDL and LDL) and plasma adipokines (leptin and adiponectin) levels.

### 2.4. Biochemical Measurements

After overnight fasting, blood collection was performed following the initial assessment (week-0) and at the end of the study (week-12) in ethylenediaminetetraacetic acid (EDTA) or heparin as appropriate. These blood samples were immediately stored at 4 °C and centrifuged for 15 min at 1000 g at 2–8 °C for 30 min. Plasma samples were aliquoted in pyrogen-free tubes and stored at −80 °C. Blood biochemistry parameters, including FBG, HbA1c and lipid levels were analyzed via the certified routine biochemistry laboratory service. The plasma leptin and adiponectin levels were determined using a sandwich ELISA kit (LINCO, Research, Saint Charies, MO, USA).

### 2.5. Gut Permeability

Biomarkers of gut permeability (ZO-1) and LPS were measured using commercial kits according to the manufacturers’ instructions. Serum LPS was assessed using a Pierce™ Limulus Amoebocyte Lysate chromogenic endotoxin quantification kit (Thermo Fisher, Sydney, NSW, Australia). The EDTA-plasma ZO-1 level was measured with a human haptoglobin ELISA kit (Abcam^®^, Sydney, NSW, Australia).

### 2.6. Inflammation

Biomarkers of inflammation were determined in serum. Serum hsCRP was measured with a human hsCRP ELISA kit (OriGene, Rockville, MD, USA). Serum tumor necrosis factor alpha (TNF-α), interleukin 6 (IL-6), interleukin 1 beta (IL-1β) and interleukin 10 (IL-10) were quantified with an ELISA kit (Thermo Fisher, Sydney, NSW, Australia) according to the manufacturers’ instructions.

### 2.7. Fecal Analysis

Stool samples were collected following the initial assessment (week-0) and at the end of the study (week-12) using a stool specimen collection kit. This collection kit contained an instruction book for the stool sample collection and transportation, ice packs, gloves, a sterile container, a sealed plastic pouch, a cool box and an AnaeroGen™ Compact Sachet, which preserves the microbiological characteristics of the sample for 72 h. The containers were stored at 4 °C. The fecal sample was analyzed by matrix-assisted laser desorption ionization–time of flight (MALDI-TOF) mass spectrometry within 24–48 h after collection. In addition, a 1 g sample was stored at −80 °C prior experiment for fecal microbiota. The fecal samples were extracted by QIAamp PowerFecal DNA/RNA kit (QIAGEN, Hidden, Germany). Fecal microbiome dataset was normalized by the total number of reads in each sample to remove potential biases related to different sequencing depth by Omics Sciences and Bioinformatics Center (OMICs, Chulalongkorn University, Thailand) using the Illumina MiSeq platform next generation sequencing system (Themo Fisher, Sydney, NSW, Australia). Sequencing data were processed using a quantitative analysis of fecal microbial ecology (QIIME II). Original sequencing reads that perfectly matched the barcode were assigned to the corresponding samples and identified as valid sequences. Low-quality sequences were filtered to remove.. After chimera detection, the remaining high-quality sequences were clustered into OTUs with 97% sequence identity. The default parameters were used to select the representative sequence from each OTU. OTUs with a total content of less than 0.001% in all samples were discarded to minimize sequencing depth differences across samples. 

Short chain fatty acids (SCFAs) were measured using high-performance liquid chromatography (HPLC) according to the modified method of Nuntawat et al. (2019). Briefly, 1 g stool samples were homogenized in 0.15 mM sulfuric acid, pH 7, mixed and centrifuged at 10,000 g for 10 min at 4 °C. The supernatant was collected and filtered through a 0.22 um nylon syringe filter. The samples were analyzed with a Shimadzu-HPLC system using Shodex SUGAR SH1011 (SHOWA DENKO K.K., Tokyo, Japan). SCFA concentrations were quantified by comparing with the standard curve and the results were expressed as μmol/g sample [20].

### 2.8. Statistical Analysis

Data were presented as mean ± SD. After testing for normality of distribution, the characteristics and biochemical variables at the beginning of the study were compared among the two groups using independent t-test or Wilcoxon rank sum test, as appropriate. Differences in sex, education, smokers and alcoholics were evaluated by Fisher’s exact test. A paired t-test or Wilcoxon signed rank test were used to determine the treatment effects within group difference. Linear regression model was used to assess the treatment effects on study outcomes among the two groups after adjusting for the confounding parameters including age, sex, education, smoker, alcoholic, BMI and the baseline biochemical parameters. A *p*-value of less than 0.05 was considered statistically significant. Data analysis was performed using STATA Statistical Software version 15.1 (Brazos County, TX, USA).

## 3. Results

A total of 50 T2DM patients were screened for eligibility and enrolled in this present randomized, double-blind, placebo-controlled clinical trial. All participants were randomly allocated into two groups (*n* = 25/group) receiving either a placebo, corn starch 10 mg/day or probiotics, *L. paracasei* HII01, at a dose of 50 × 10^9^ CFU/day for 12 weeks. Seven patients in the placebo (*n* = 7) and probiotics (*n* = 7) groups dropped out due to personal reason. Hence, 36 participants (72%) finished the study per protocol, reducing the power from 80 to 75.79. There were no statistically significant differences between the two groups regarding any of the baseline characteristics and biochemical parameters at the beginning of the study (Table S1 (Supplementary File)). There were no seriously adverse effects or symptoms among the participants and they all demonstrated good compliance. The medication of individual participants did not change during the study. (Table S2 (Supplementary File)).

Demographic characteristics of the participants are shown in Table 1. There was no significant difference in age, sex, education, smokers, alcoholics and BMI between placebo and probiotic group at the beginning of the study.

### 3.1. The Effect of L. paracasei HII01 Supplementation on Blood Biochemical Parameters

Table 2 illustrates the biochemical parameters assessment of the two groups at baseline and at the end of the study with a comparison of within-group changes. At the end of the 12 weeks of intervention, there was no significant change in the level of all parameters detected in the placebo group compared to baseline. Interestingly, the FBG level in probiotic group at the end of the study significantly reduced compared to baseline (*p* < 0.05). In addition, the FBG level in probiotic group was also significantly reduced when comparing with placebo group at the end of study. (*p* < 0.05) (Table 3). As is well-known, adipokine plays an important role in regulating glucose metabolism. Thus, we next evaluated the plasma level of two adipokines, namely adiponectin and leptin. The results showed that there were no significant differences in either plasma adiponectin or leptin levels within the groups compared with the baseline values (Table 2). Regardless, the plasma leptin and adiponectin levels likely improve in the probiotics group. However, between-group comparisons showed no significant changes in these adipokines levels at the end of the intervention as shown in Table 3.

As can be seen in Table 2, the changes of the plasma TG and cholesterol levels were not observed in both placebo and probiotic group compared with the baseline and between-group comparisons. However, the plasma LDL level significantly decreased in the probiotic group between baseline and the end of the study (*p* < 0.05). A significant increase in the plasma HDL level was also exhibited in the *L. paracasei* HII01-treated group compared with the baseline (*p* < 0.05). In addition, we detected a significant decrease of LDL level together with increase HDL level in the probiotics group compared to placebo (*p* < 0.05) (Table 3). These findings may suggest that dyslipidemia in T2DM individuals was improved after the administration of *L. paracasei* HII01 for 12 weeks.

### 3.2. The Effect of L. paracasei HII01 Supplementation on the Level of ZO-1 and Inflammation Parameters

To evaluate the underlying mechanisms involving the possible beneficial effects of *L. paracasei* HII01 in improving gut permeability and endotoxemia, the levels of the tight junction protein, LPS, and pro-inflammatory cytokines were investigated and are presented in Table 4. Within group comparison showed that no differences were observed in all parameters in the placebo group. Interestingly, a significant decrease of the plasma LPS level was found in the probiotic *L. paracasei* HII01 group when compared with baseline (*p* < 0.05). Additionally, the levels of TNF-α and IL-6 at the end of the study had significantly decreased in the probiotic group compared to baseline (*p* < 0.05). Likewise, participants in the probiotic group also showed a significant reduction in the LPS, TNF-α and IL-6 levels after 12 weeks of intervention compared to participants in the placebo group (*p* < 0.05) (Table 5).

In this study, we also measured the plasma level of hsCRP, which is a biomarker of inflammation. There was no significant alteration in the plasma hsCRP level when compared with baseline in the placebo group. Remarkably, *L. paracasei* HII01 supplementation for 12 weeks in T2DM individuals significantly reduced the plasma hsCRP level compared with baseline (*p* < 0.05) (Table 4). Likewise, the reduction of hsCRP level was found in probiotic group compared with placebo group (*p* < 0.05) (Table 5). Furthermore, IgA, which is an antibody that plays a major role in the immune function, significantly increased in the probiotic group when compared with the baseline (*p* < 0.05) (Table 4). Together, these findings suggested that supplementation with *L. paracasei* HII01 attenuated endotoxemia and systemic inflammation in T2DM patients.

### 3.3. The Effect of L. paracasei HII01 Supplementation on the Level of SCFAs

It has been well established that gut microbiota also influences glucose metabolism partly via the production of SCFAs. Therefore, lactic, propionic, butyric and acetic acids, which are the most important SCFAs that affect glycemic control, were determined (Table 6). Interestingly, the results of the probiotic group demonstrated that the level of SCFAs, i.e., lactic, propionic, acetic and butyric acid, significantly increased within the group when compared with baseline (*p* < 0.05), while no significant difference was observed in the placebo group (*p* > 0.05) (Table 6). There were no significant differences between the two groups in those SCFAs levels at the end of the study. These findings indicated that administration of *L. paracasei* HII01 increased the level of SCFAs and may have contributed to the regulation of glucose metabolism in T2DM patients.

### 3.4. The Effect of L. paracasei HII01 Supplementation on Microbial Diversity

**Figure 2:**
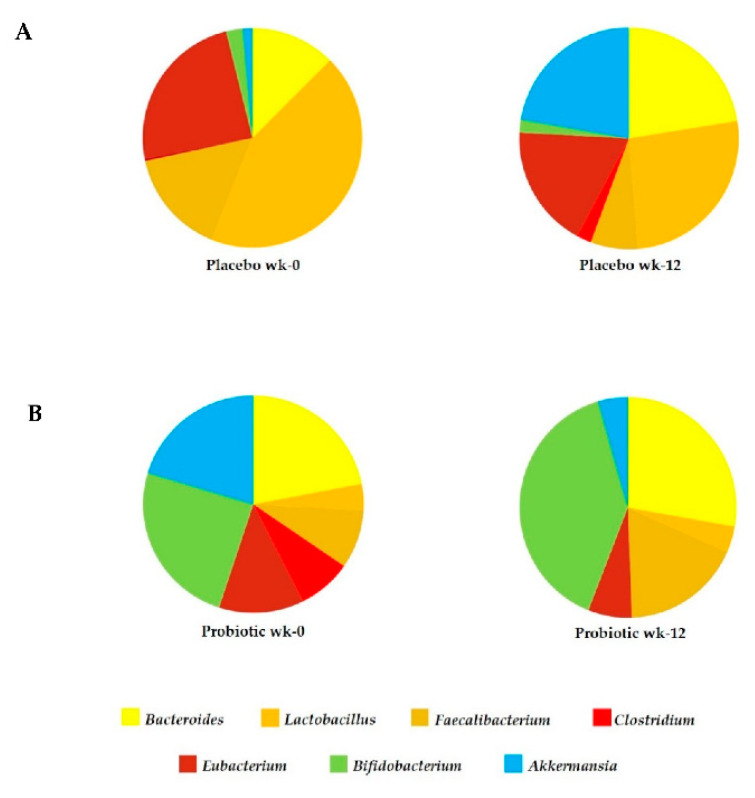
Seven genera: *Akkermansia*, *Bifidobacterium*, *Eubacterium*, *Clostridium*, *Faecalibacterium*, *Lactobacillus*, and *Bacteroides* dominated the majority of OTUs. 16S rRNA gene sequences were used for identification. The relative abundance of bacteria in feces of placebo group (**A**) and probiotic group (**B**) at baseline and at the end of the study.

The relative abundance OTUsplot of bacterial genus diversity in feces is illustrated in Figure 2. In comparison with baseline, sequences were distributed among seven genera including *Akkermansia*, *Bifidobacterium*, *Eubacterium*, *Clostridium*, *Faecalibacterium*, *Lactobacillus* and *Bacteroides*. The abundance percentage level of beneficial bacteria, such as *Lactobacillus*, *Faecalibacterium* and *Bifidobacterium* markedly increased after probiotic *L. paracasei* HII01 supplementation. There was no significantly higher difference in the *Lactobacillus* composition. In contrast, the proportion of genus *Bifidobacterium*, *Bacteroides* and *Faecalibacterium* significantly increased in the probiotic *L. paracasei* HII01 supplementation group compared to placebo group (*p* < 0.05, 95% confidence interval). *Bifidobacterium* was the most altered among those fecal bacteria, varying from 17 to 29%, after *L. paracasei* HII01 administration. Notably, the pathogenic *Clostridium* abundance was increased only in the placebo group and this was not observed in the probiotic treatment group (Figure 2). These outcomes suggested that *L. paracasei* HII01 administration effectively enhanced some beneficial bacteria as well as reduced the pathogenic bacteria in T2DM patients.

## 4. Discussion

The main findings of this study were that intervention with *L. paracasei* HII01 for 12 weeks improved glycemia in T2DM patients. Importantly, *L. paracasei* HII01 supplementation was able to affect endotoxemia and improve inflammatory cytokines. The modifying fecal microbiome seemed to play a role in the facilitation and extent of these beneficial effects.

The improvement in glycemic control and other aspects of diabetes that were found in this present trial is in line with other studies. Supplementation with fermented milk with the *L. acidophilus*, *L. casei* and *Bifidobacteria* for 8 weeks reduced the level of FBG and HbA1c compared with the control group in T2DM patients in a randomized, double-blind, placebo-controlled clinical trial [21]. Sivieri K. and colleagues (2012) reported that daily consumption of 4 × 10^8^ CFU/100 mL of *L. acidophilus*, 4 × 10^8^ CFU/100 mL of *B. bifidum* and 1 g/100 mL of fructooligosaccharides for 30 days resulted in significantly decreased FBG in T2DM individuals [22]. Moreover, it has been reported that consumption of capsules containing 10^8^ CFU of *L. casei* for 8 weeks significantly reduced FBG and insulin resistance in T2DM patients [23]. In contrast, Horvath A. et al. (2020) recently reported that there were no changes in glucose metabolism or mixed meal tolerance test responses in diabesity patients receiving a multispecies probiotic and a prebiotic for 6 months [24]. Possible explanations for the contradictory results from clinical studies are manifold and related to, among other things, individual participants, probiotic formulations, and the concentration and duration of the intervention. However, the precise mechanisms of the beneficial effects of probiotics on glucose metabolism remain unclear. Previous studies suggested that some kinds of probiotics could change, in particular, the composition of gut microbiota (i.e., increase the good bacteria and decrease the pathogenic bacteria), leading to reduced gut dysbiosis [25,26,27]. It has been shown that an increase in the level of the pathogenic bacteria *Clostridium,* together with a reduction in the level of beneficial bacteria, namely *Bacteroides*, *Bifidobacterium* and *Actinobacteria*, was experienced by T2DM patients [28,29]. Additionally, an association between the improvement of FBG and higher levels of *Bacteroides*, *Bifidobacterium* and *Feacalibacterium* has been reported [30,31]. In this study, the genus level changes of gut microbiota were explored. Even the abundance of those bacteria at baseline was not the same compared between the two groups but the resulted revealed that the percentage abundance of *Bacteriodes*, *Lactobacillus*, *Faecalibacterium* and *Bifidobacterium* increased after probiotic *L. paracasei* HII01 supplementation for 12 weeks in T2DM patients. Interestingly, the level of *Clostridium* evidently decreased in the probiotic group, while the opposite result was found in the placebo group. Ducarmon and coworkers (2019) reported the probiotic bacteria, such as *Lactobacillus* and *Bifidobacterium* had an effect against pathogenic bacteria via reducing the gut pH leading to inhibition of the growth of pathogenic bacteria or increased mucin secretion leading to increased mucus layer and prohibiting the colonization of pathogenic bacteria [32]. Although the probiotic *L. paracasei* HII01 was supplemented, the abundance OTUs of *Lactobacillus* was lower than the abundance of *Bifidobacterium* at week 12 in feces of T2DM patients. *Lactobacillus* strain was fed to human volunteers and then fecal microbiota were examined after 12 weeks of administration. While the administration continued, total *Lactobacillus* (flora and the administered strains) excretion slightly increased in feces but total *Bifidobacterium* was greatly increased. The results were that two probiotic bacterial genera can survive the passage through the gastrointestinal tract, but *Lactobacillus* do not colonize the gastrointestinal tract to a significant extent. Moreover, the fecal microbiome OTUs was determined at weeks 0 and 12 but the dynamic change of probiotic *Lactobacillus* colonization might increase in shorter term than 12 weeks. Further work on the kinetic changes of gut microbiota should be classified. However, the colonization of administered *Lactobacillus* may be unnecessary to achieve functional properties in probiotic therapy and promote symbiotic growth for good bacterial *Bifidobacterium*. Besides, the present study found that the participant’s microbiota had low diversity, was rich in members of *Lactobacillus* and *Bifidobacterium*, and had low numbers of *Clostridium*. There are many factors for reducing the abundance of *Clostridium* such as dietary intake, host genetics, age, residence area or medication [33]. Additionally, the evidence informed that some antidiabetic medications could be against the colonization of *Clostridium*. A previous study reported that treatment with metformin had a protective effect against the colonization of *Clostridium difficile* in diabetic patients [34]. On the other hand, the reduction of Gram-negative bacteria, for example *Bacteroides*, relating to their increased number of deaths, was noted in diabetic patients [4].

Gut microbiota may be involved in insulin resistance and type 2 diabetes mellitus through several probable mechanisms: for example, alteration of energy homeostasis or glucose metabolism and also low-grade inflammation [8]. One common theory is that bacterial LPS derives from the outer membranes of Gram-negative bacteria, which has been known to induce metabolic endotoxemia by promoting secretion of pro-inflammatory cytokines [35]. LPS can translocate through the damaged gut barrier. Then, LPS binds to TLR4 and activates the TLR4/CD14 complex, which activates pro-inflammatory pathways [36]. Chronic exposure to pro-inflammatory cytokines such as TNF-α, IL-1β and IL-6 activates the signaling proteins that block the activation of the insulin signaling cascade in the target organs of insulin, including skeletal muscle, adipose tissue and liver, leading to hyperglycemia [37]. To support this concept, our findings clearly showed that the systemic inflammation resulting from plasma endotoxemia or LPS levels was significantly reduced in the *L. paracasei* HII0-treated group, indicating decreased translocation of bacterial products. Additionally, supplementation with *L. paracasei* HII01 for 12 weeks decreased the plasma level of inflammatory cytokines, including TNF-α, IL-6 and hsCRP when compared to their baseline values. Another probable pathway mediating the connection between gut microbiota and metabolic endotoxemia is gut permeability [4]. Our previous study demonstrated that supplementation with the *L. paracasei* HII01 for 12 weeks in T2DM rat resulted in lessening plasma levels of DX-4000-FITC, suggesting an improvement of gut barrier integrity [38]. This effect could directly ameliorate systemic endotoxemia by reducing the leakage of LPS into systemic circulation.

Gut dysbiosis could induce abnormal immune responses such as the abnormal secretion of IgA [5]. Moreover, the presence of LPS in blood circulation not only acts as a potent inflammatory mediator and influences insulin sensitivity but also disturbs the functionality of the innate immune system [39]. In the present study, we found that the IgA levels in the *L. paracasei* HII01-treated group were increased when compared to baseline. Similarly, results from an in vivo study revealed that *L. lactis* increased the IgA secretion [40]. It has also been reported that the *B. breve* increased the level of IgA, leading to an increased humoral immune response [41].

Previous articles have reported that the antidiabetic effect of probiotics may be due to microbial metabolites such as SCFAs [42]. SCFAs are fermentation products of carbohydrates or proteins by probiotic bacteria. [43]. A reduction of SCFAs levels has been detected in T2DM [44]. Importantly, the results from this study demonstrated that the administration of *L. paracasei* HII01 for 12 weeks in T2DM patients effectively increased the number of those SCFAs in fecal content compared to baseline. Several mechanisms have been proposed to explain the effects of SCFAs in the improvement of diabetes. These SCFAs are not only of importance in gut health as signaling molecules but might also enter the systemic circulation and directly affect peripheral tissues via AMPK activation and subsequently GLUT4 translocation to membrane for uptake of glucose into cells [45]. In addition, our previous study found that supplementation of *L. paracasei* HII01 alleviated hyperglycemia in diabetic rats via increasing glucose uptake by the skeletal muscle which was mediated partly by PI3K/Akt and AMPK activation [38].

In this study, there were no significant differences in the plasma TG and cholesterol levels at the end of the study. These results were consistent with the previous study demonstrating that *Lactobacillus* supplementation significantly decreased the total cholesterol and LDL levels, while no significant effects were found on the TG and HDL levels [46]. A meta-analysis of 12 randomized controlled trials found that the effects of probiotics on the lipid profile were nonsignificant [47]. The authors suggested that the levels of lipid profiles were inconclusive due to various factors such as sample sizes, subject status, age and BMI. However, *L. paracasei* HII01 supplementation significantly reduced the LDL level together with enhanced HDL level after 12 weeks of the intervention. As mentioned earlier, the SCFAs can induce AMPK activation. Our data revealed that treatment of *L. paracasei* HII01 increased the level of SCFAs. AMPK activation in the liver leads to the stimulation of fatty acid oxidation and suppression of lipogenesis through the inhibition the enzymes 3-hydroxy-3-methylglutaryl-CoA (HMG-CoA) reductase and acetyl-CoA carboxylase (ACC) [40]. Therefore, it may play a role in the regulation of lipid metabolism by probiotics.

This study has some limitations in the interpretation of our findings. Due to the methodology limitation, we did not characterize the gut microbiota at the level of species and thus, successful colonization of probiotic *Lactobacillus paracasei* HII01 in participants’ guts cannot be confirmed. Additionally, this study is relatively biased by the unequal sex distribution of the study participants. In total, 77.77% of the study participants were female, and this fact might be one of the confounding factors affecting the gut microbiome composition. In addition, dietary, physical activity assessment and other information including frequency and characteristic of feces in this study relied only on subjective reports which are not as accurate as objective methods for measuring their compliance.

In conclusion, the outcomes of this present trial firstly point to beneficial effects of *L. paracasei* HII01 supplementation in terms of glycemic improvement and other metabolic variables by favorably modifying the gut microbiota and subsequently ameliorating endotoxemia, which suggests a potential role of probiotic *L. paracasei* HII01 as a commercial probiotic product for an add-on treatment in subjects with T2DM.

## Figures and Tables

**Table 1:** General characteristics of the type 2 diabetes participants.

Characteristics	Placebo (*n* = 18)	Probiotic (*n* = 18)	*p*-Value
Age (year)	61.78 ± 7.73	63.50 ± 5.94	0.459 ^a^
Sex			0.228 ^b^
Male	2 (11.11)	6 (33.33)	
Female	16 (88.89)	12 (66.67)	
Education			1.000 ^b^
No education	2 (11.11)	3 (16.67)	
Primary	14 (77.78)	13 (72.22)	
Secondary	2 (11.11)	2 (11.11)	
Smokers			0.486 ^b^
No	18 (100)	16 (88.89)	
Yes	0 (0)	2 (11.11)	
Alcoholic			1.000 ^b^
No	17 (94.44)	18 (100)	
Yes	1 (5.56)	0 (0)	
BMI (kg/m^2^)	23.05 ± 2.60	23.22 ± 2.72	0.852 ^a^

Data are mean ± SD. ^a^ *p*-value obtained from independent t-test, ^b^ *p*-value obtained from Fisher’s exact test.

**Table 2:** Blood levels of FBG, HbA1c, adiponectin, leptin and lipid profiles at baseline and at the end of the study.

Parameters	Placebo (*n* = 18)			Probiotic (*n* = 18)		
	Wk-0	Wk-12	*p*-Value	Wk-0	Wk-12	*p*-Value
FBG (mg/dL)	139.29 ± 49.77	149.12 ± 47.42	0.208 ^a^	129.18 ± 34.52	109.35 ± 15.56	0.005 ^a^
HbA1c (%)	6.64 ± 1.44	6.73 ± 1.29	0.837 ^a^	7.05 ± 1.85	6.46 ± 1.49	0.145 ^a^
Adiponectin (ng/mL)	27.70 ± 7.26	24.03 ± 12.52	0.465 ^b^	23.40 ± 8.75	30.73 ± 18.40	0.763 ^b^
Leptin (ng/mL)	15.56 ± 13.56	26.46 ± 8.49	0.095 ^b^	15.74 ± 9.51	14.60 ± 8.70	0.345 ^b^
TG (mg/dL)	147.78 ± 71.32	159.50 ± 67.87	0.581 ^a^	153.27 ± 55.46	144.53 ± 58.68	0.560 ^a^
Cholesterol (mg/dL)	190.83 ± 43.22	184.94 ± 46.65	0.314 ^a^	188.59 ± 34.21	182.88 ± 30.38	0.453 ^a^
LDL (mg/dL)	102.11 ± 32.60	90.61 ± 16.06	0.106 ^a^	109.24 ± 38.44	81.47 ± 33.53	<0.00 ^a^
HDL (mg/dL)	62.87 ± 9.83	68.67 ± 13.99	0.145 ^a^	62.06 ± 14.84	72.06 ± 21.34	0.026 ^a^

Data are mean ± SD. Difference between baseline and the end of study for the within group comparisons. ^a^ *p*-value obtained from paired t-test, ^b^ *p*-value obtained from Wilcoxon signed rank test.

**Table 3:** The differentiation of blood levels of FBG, HbA1c, adiponectin, leptin and lipid profiles between *L. paracasei* HII01 treatment and placebo group at the end of study.

Comparison to Placebo at Week-12			
Probiotic Group			
Parameters	Coef.	95% Confidence Interval	*p*-Value
FBG (mg/dL)	−37.16	(−60.35, −13.97)	0.004
HbA1c (%)	−1.50	(−4.62, 1.62)	0.252
Adiponectin (ng/mL)	6.03	(22.64, 10.59)	0.371
Leptin (ng/mL)	−7.44	(−26.02, 11.15)	0.292
TG (mg/dL)	−45.35	(−119.26, 28.55)	0.211
Cholesterol (mg/dL)	−17.35	(−40.33, 5.63)	0.128
LDL (mg/dL)	−18.54	(−34.63, −2.44)	0.0026
HDL (mg/dL)	14.53	(−0.29, 28.77)	0.046

Data are mean ± SD. Significant difference between groups at the end of study. *p*-values obtained from linear regression after adjusting for baseline characteristics and baseline of the corresponding parameter. Coefficient (Coef.) is defined as the difference in outcome measures between probiotic and placebo groups.

**Table 4:** Blood levels of ZO-1 and inflammation parameters at baseline and at the end of the study.

Parameters	Placebo (*n* = 18)			Probiotic (*n* = 18)		
	Wk-0	Wk-12	*p*-Value	Wk-0	Wk-12	*p*-Value
ZO-1 (ng/mL)	1.80 ± 0.86	1.74 ± 0.77	0.455 ^a^	1.61 ± 0.53	1.45 ± 0.63	0.204 ^a^
LPS (pg/mL)	72.38 ± 40.67	69.36 ± 24.91	0.638 ^b^	92.05 ± 35.95	51.07 ± 20.26	0.002 ^b^
TNF-α (ng/mL)	11.75 ± 3.01	9.62 ± 1.37	0.252 ^a^	11.91 ± 0.72	6.16 ± 0.90	0.009 ^a^
IL-1β (ng/mL)	7.26 ± 2.49	6.17 ± 1.42	0.345 ^b^	8.21 ± 3.52	5.46 ± 3.24	0.109 ^b^
IL-6 (ng/mL)	11.84 ± 1.78	10.76 ± 4.91	0.549 ^a^	11.01 ± 1.91	7.85 ± 2.92	0.033 ^a^
IL-10 (ng/mL)	1.27 ± 0.15	6.04 ± 5.43	0.068 ^b^	1.12 ± 0.05	10.58 ± 0.1	0.180 ^b^
IgA (ng/mL)	660.68 ± 262.60	679.65 ± 338.66	0.867 ^a^	526.24 ± 249.61	707.02 ± 265.37	0.027 ^a^
hsCRP (mg/L)	0.0160 ± 0.0069	0.0158 ± 0.0059	0.950 ^a^	0.0143 ± 0.0024	0.0124 ± 0.0026	0.032 ^a^

Data are mean ± SD. Difference between baseline and the end of study for the within group comparisons. ^a^ *p*-value obtained from paired *t*-test, ^b^ *p*-value obtained from Wilcoxon signed rank test.

**Table 5:** The differentiation of blood levels of ZO-1 and inflammation parameters between *L. paracasei* HII01 treatment and placebo at the end of study.

Comparison to Placebo at Week-12			
Probiotic			
Parameters	Coef.	95% Confidence Interval	*p*-Value
ZO-1 (ng/mL)	−0.30	(−0.69, −0.09)	0.124
LPS (pg/mL)	−26.46	(−46.55, −6.37)	0.020
TNF-α (ng/mL)	−3.16	(−5.50, −0.82)	0.023
IL-1β (ng/mL)	−2.13	(−5.72, 1.46)	0.125
IL-6 (ng/mL)	−4.12	(−5.68, −2.56)	0.001
IL-10 (ng/mL)	5.61	(−87.25, 98.47)	0.060
IgA (ng/mL)	257.62	(663.69, −148.45)	0.188
hsCRP (mg/L)	−0.004	(−0.008, −0.001)	0.026

Data are mean ± SD. Significant difference between groups at the end of study. *p*-values obtained from linear regression after adjusting for baseline characteristics and baseline of the corresponding parameter. Coefficient (Coef.) is defined as the difference in outcome measures between probiotic and placebo groups.

**Table 6:** The levels of SCFAs in feces at baseline and at the end of the study.

Parameters	Placebo (*n* = 18)			Probiotic (*n* = 18)		
	Wk-0	Wk-12	*p*-Value	Wk-0	Wk-12	*p*-Value
Lactic acid (μmol/g)	22.60 ± 19.57	40.73 ± 25.80	0.068 ^b^	20.46 ± 16.08	51.00 ± 27.96	0.028 ^b^
Propionic acid (μmol/g)	42.83 ± 17.91	211.93 ± 30.28	0.109 ^b^	49.86 ± 94.36	307.64 ± 104.55	0.012 ^b^
Acetic acid (μmol/g)	359.40 ± 75.80	405.18 ± 71.08	0.217 ^a^	343.84 ± 46.56	512.54 ± 79.57	0.003 ^a^
Butyric acid (μmol/g)	51.46 ± 13.68	162.45 ± 57.14	0.109 ^b^	48.70 ± 29.76	181.80 ± 147.48	0.012 ^b^

Data are mean ±SD. Difference between baseline and the end of study for the within group comparisons. ^a^ *p*-value obtained from paired t-test, ^b^ *p*-value obtained from Wilcoxon signed rank test.

## Supplementary Materials

The following are available online at https://www.mdpi.com/article/10.3390/foods10071455/s1. Table S1: Baseline biochemical parameters at the beginning of the study; Table S2: Oral antidiabetic drugs used by participants.
