# Supplementary material for: Influence of Lactobacillus paracasei HII01 Supplementation on Glycemia and Inflammatory Biomarkers in Type 2 Diabetes: A Randomized Clinical Trial
Source: Foods. 2021 Jun 23;10(7):1455. doi: 10.3390/foods10071455 (PMC8303256; doi:10.3390/foods10071455)
Supplement: Supplementary file 1 [file foods-10-01455-s001.zip › foods-1132254-supplementary.pdf]

Table S1 Baseline biochemical parameters at the beginning of the study.

Variable	Placebo (n=18)	Probiotic (n=18)	*P* value
BMI	23.05 ± 2.60	23.22 ± 2.72	0.852 [a]
BUN (mg/dL)	19.45 ± 9.24	8.87 ± 9.04	0.850 [a]
Creatinine (mg/dL)	1.46 ± 0.57	1.60 ± 0.70	0.539 [a]
AST (IU/L)	21.94 ± 8.10	27.50 ± 36.96	0.392 [b]
ALT (IU/L)	22.33 ± 12.39	19.89 ± 16.58	0.168 [b]
ALP (IU/L)	100.39 ± 20.86	98.61 ± 28.99	0.834 [a]
FBG (mg/dL)	139.29 ± 49.77	129.18 ± 34.52	0.496 [a]
HbA1c (mg%)	6.64 ± 1.44	7.05 ± 1.85	0.468 [a]
Cholesterol (mg/dL)	190.83 ± 43.22	188.59 ± 34.21	0.866 [a]
Triglyceride (mg/dL)	147.78 ± 71.32	153.27 ± 55.46	0.810 [a]
HDL (mg/dL)	62.87 ± 9.83	62.06 ± 14.84	0.859 [a]
LDL (md/dL)	102.11 ± 32.60	109.24 ± 38.44	0.558 [a]
Leptin (ng/mL)	15.56 ± 13.56	15.74 ± 9.51	0.831 [b]
Adiponectin (ng/mL)	27.70 ± 7.26	23.40 ± 8.75	0.441 [a]
IgA (ng/mL)	660.68 ± 262.60	526.24 ± 249.61	0.150 [a]
LPS (pg/mL)	72.38 ±40.67	92.05 ± 35.95	0.094 [b]
ZO-1 (ng/mL)	1.80 ± 0.86	1.61 ± 0.53	0.451 [a]
hsCRP (ml/L)	0.016 ± 0.007	0.014 ± 0.002	0.385 [a]
Lactic acid (µmol/g)	22.60 ± 19.57	20.46 ± 16.08	0.831 [b]
Acetic acid (µmol/g)	359.40 ± 75.80	343.84 ± 46.56	0.695 [a]
Propionic acid (µmol/g)	42.83 ± 17.91	49.86 ± 94.36	0.103 [b]
Butyric acid (µmol/g)	51.46 ± 13.68	48.71 ± 29.76	1.000 [b]
IL-6 (ng/mL)	11.73 ± 1.66	10.07 ± 3.05	0.169 [a]
IL-10 (ng/mL)	1.27 ± 0.15	1.12 ± 0.05	0.232 [a]
IL-1β (ng/mL)	7.26 ± 2.49	8.21 ± 3.52	0.650 [a]
TNF-α (ng/mL)	11.75 ± 3.01	11.91 ± 0.72	0.932 [a]

Data are mean ± SD. [a] p-value obtained from independent t-test, [b] *p*-value obtained from Wilcoxon rank sum test.

Table S2 Oral anti-diabetic drugs used of participants.

Anti-diabetic drugs	Placebo group	Probiotic group
metformin	18	18
sulfonylurea	10	8
alpha-glucosidase inhibitors	4	2

n=18/group

All participants in both groups received metformin (100%)
Some participants received metformin combine with sulfonylurea or alpha-glucosidase inhibitor.
Placebo group; metformin combines with sulfonylurea = 55.5%
metformin combines with alpha-glucosidase inhibitor = 22.2%
Probiotic group; metformin combines with sulfonylurea = 44.4%
metformin combines with alpha-glucosidase inhibitor = 11.1%
